# Draft Genome Sequence of Staphylococcus aureus Strain HD1410, Isolated from a Persistent Nasal Carrier

**DOI:** 10.1128/genomeA.00411-18

**Published:** 2018-05-10

**Authors:** Dennis Nurjadi, Sébastien Boutin, Alexander Dalpke, Klaus Heeg, Philipp Zanger

**Affiliations:** a Department of Infectious Diseases, Medical Microbiology and Hygiene, Heidelberg University Hospital, Heidelberg, Germany; b Institute of Public Health, Heidelberg University Hospital, Heidelberg, Germany

## Abstract

We report here the draft genome sequence of a Staphylococcus aureus strain isolated from the nares of an 18-year-old female healthy persistent-carrier individual, and it was used to investigate S. aureus-specific immune responses in colonized and noncolonized individuals.

## GENOME ANNOUNCEMENT

Staphylococcus aureus colonization is an important reservoir for S. aureus in the human population. In patients, persistent colonization increases the risk of acquiring S. aureus infections (1). The mechanisms which promote colonization are multifactorial and yet not fully elucidated. To experimentally mimic the physiological interaction between S. aureus and the host’s immune response, viable bacteria are crucial. Current knowledge suggests that (tolerogenic) immune responses toward this pathogen are strain specific (2). Therefore, the suitability of using lab strains for immunological studies is doubtful.

We isolated an S. aureus strain, HD1410, from an 18-year-old Caucasian female with persistent nasal carriage in June 2009. She was identified from a cohort of 603 volunteers subjected to phenotyping for S. aureus nasal carriage by four consecutive nasal swabs (3). Persistent colonization was defined as culture positivity for S. aureus in all four swabs, which were taken over a period of 107 days. The sample collection was approved by the local institutional review board. Viable S. aureus HD1410 was then used to stimulate whole blood and study the expression profile of T-cell cytokines (4) and Toll-like receptor 9 (TLR9) (5) in S. aureus persistent carriers and noncarriers.

S. aureus genomic DNA was extracted using the DNeasy blood and tissue kit (Qiagen GmbH, Germany) with an additional prior lysis with lysostaphin (Genaxxon Bioscience, Germany). A standard genomic library was prepared from the extracted DNA and sequenced with the Illumina HiSeq platform (2 × 150 bp paired end) by GATC Biotech AG (Constance, Germany). Raw sequences were trimmed for quality using Sickle 1.33 (parameters, q > 30; 1 > 45) (6). The cleaned sequences were then assembled using SPAdes 3.10.0 (7). Contigs obtained from the assembly were therefore curated for length (>500 bp) and coverage (>10×) to ensure no errors and contamination in the draft genome. The contigs were then annotated using Prokka 1.12 (based on Genetic Code Table 11) (8) and the NCBI Prokaryotic Genome Annotation Pipeline.

The multilocus sequence typing (MLST), VirulenceFinder, ResFinder, and PlasmidFinder (http://genomicepidemiology.org) databases were used to determine the sequence type, virulence genes, resistance, and plasmid type, respectively. The isolate belongs to clonal complex 30 (CC30)/sequence type 34 (ST34) with *spa* type t136. Phenotypic resistance to penicillin was confirmed by the presence of the penicillinase gene *blaZ*. No other phenotypic or genotypic resistances could be detected.

The assembled genome contains 38 contigs, for a total length of approximately 2.8 Mb, GC content of 32.7%, and an average coverage of 241×, which were estimated using SPAdes assembly. A total of 2,954 genes were found containing 2,770 coding sequences (CDSs).

The toxin genes encoding enterotoxins G, H, I, M, N, O, and U (*seg*, *seh*, *sei*, *smi*, *sen*, *seo*, and *seu*, respectively) and toxic shock toxin (*tst-1*) were present. Two plasmids carrying resistance and virulence genes *rep16*, with the DUF536 domain-containing protein, and *rep5*, with a replication-initiating protein, could be detected.

### Accession number(s).

The draft genome sequence has been deposited in the DDBJ/EMBL/GenBank database under the accession number NXFH00000000.
